# A Short Note on Aberrant Responses Bias in Item Response Theory

**DOI:** 10.3389/fpsyg.2019.00043

**Published:** 2019-01-31

**Authors:** Bing Jia, Xue Zhang, Zhemin Zhu

**Affiliations:** ^1^Teacher Training Center, Beihua University, Jilin City, China; ^2^China Institute of Rural Education Development, Northeast Normal University, Changchun, China; ^3^School of Education Science, Beihua University, Jilin City, China

**Keywords:** maximum likelihood estimation, ABIAS, |ABIAS| method, aberrant response, bias

## Abstract

Item response models often cannot calculate true individual response probabilities because of the existence of response disturbances (such as guessing and cheating). Many studies on aberrant responses under item response theory (IRT) framework had been conducted. Some of them focused on how to reduce the effect of aberrant responses, and others focused on how to detect aberrant examinees, such as person fit analysis. The purpose of this research was to derive a generalized formula of bias with/without aberrant responses, that showed the effect of both non-aberrant and aberrant response data on the bias of capability estimation mathematically. A new evaluation criterion, named aberrant absolute bias (|ABIAS|), was proposed to detect aberrant examinees. Simulation studies and application to a real dataset were conducted to demonstrate the efficiency and the utility of |ABIAS|.

## Introduction

Item response theory (IRT) is a statistical method based on an examinee's response to explain his/her ability. Thus, the classical estimate of ability in IRT is highly sensitive to response disturbance (Magis, 2014). It can return a strongly biased estimation of true underlying ability when the responses are aberrant. The aberrant responses are often strange and different than expected. In fact, a response inconsistent with expectation is said to be aberrant (Clark, 2010). There are various sources of aberrant responses. Meijer (1996) proposed seven examinee behaviors that could cause aberrant responses: sleeping, guessing, cheating, plodding, alignment errors, extreme creativity, and deficiency of sub-abilities. For example, if an examinee chose the right answer by randomly guessing on a multiple-choice item, the test score might be inflated, leading to a higher than the actual impression of the respondent.

Aberrant responses occur when the observed response patterns are incongruous with the expected ones (Meijer, 1996; Meijer et al., 1996; Meijer and Sijtsma, 2001), which may jeopardize measurement accuracy among respondents and invalidate the use of IRT. Aberrant responses had been explored in IRT literature. Under the IRT framework, aberrant responses were addressed through (i) methods based on response times (RTs), such as classical and Bayesian checks in computerized adaptive testing (CAT; van der Linden et al., 1999; van der Linden and van Krimpen-Stoop, 2003; van der Linden, 2008); (ii) methods without response times, such as person fit analysis to identify aberrant examinees (Meijer and Sijtsma, 2001; Meijer, 2003; Emons, 2009), and weight robust estimation to reduce the influence of aberrant responses on ability estimation (Wainer and Wright, 1980; Schuster and Yuan, 2011; Magis, 2014).

Under IRT framework, RTs can be used as collateral information to analyze response data with/without abnormality. For example, time pressure can sometimes cause the high ability examinee be assigned to more difficult items (Wainer and Wang, 2007). However, the application of RTs is restricted in computer environment.

Person-fit statistics which can be used in both computer and non-computer environments are designed to identify examinees with aberrant item response patterns (Karabatsos, 2003). Karabatsos (2003) compared 36 person-fit indicators under different testing conditions, and found that *H*^*T*^ (Sijtsma, 1986) statistic, which was a non-parametric statistic, was the best indicator to detect aberrant examinees. However, the most widely used parametric person fit indicators are *l*_*z*_ (Drasgow et al., 1985) and CUSUM-based (cumulative sum based) indicators (Meijer, 2002).

The *l*_*z*_ is used to quantify persons' adherence to the corresponding IRT model, and large negative value of *l*_*z*_ indicates aberrant responses (Meijer and Sijtsma, 2001; Meijer, 2003). The CUSUM-based technique (Bradlow et al., 1998; van Krimpen-Stoop and Meijer, 2000, 2001, 2002; Bradlow and Weiss, 2001; Meijer, 2002) provides information about what occurred to each item during the answering process to detect a local misfit. Meijer (2002) found that CUSUM could provide more information about local misfit than the *l*_*z*_ index. However, if one or more of the parameters are unknown, the power of CUSUM may be unsatisfactory (Csorgo and Horvath, 1997; Chen and Gupta, 2012).

On the other hand, instead of detecting aberrant behavior, weight robust estimation can be used to reduce the bias in estimating by weighting. Wainer and Wright (1980) were first to propose a robust approach in estimating ability in IRT. Mislevy and Bock (1982), Schuster and Yuan (2011) improved Wainer and Wright's approach by introducing different smoother weight functions. The estimation effect of the new method is more accurate.

When an examinee has aberrant responses, the ability estimate based on the whole responses is not the “true” ability estimate, that is because the ability estimate, $\hat{\theta }$ will be affected by aberrant responses (Magis, 2014). Generally speaking, if one's responses are non-aberrant, the responses will point to the “true” ability estimate. In contrast, if the responses contain some aberrant responses, the aberrant ones will point to the aberrant ability estimate. Hence, the bias of the ability estimations may variate with the ratio of aberrant responses to the whole responses. This provides a new direction to detect aberrant examinees.

**Figure 1:**
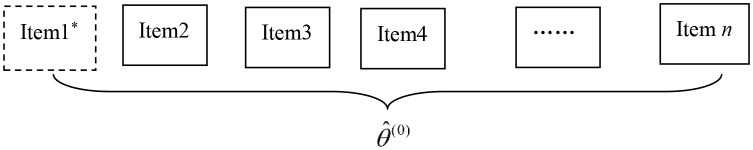
Estimation of ability by MLE, ${\hat{\theta }}^{\left(0\right)}$ in aberrant responses.

In a simple way, for one examinee, suppose the first response is aberrant, and others are non-aberrant. ${\hat{\theta }}^{\left(0\right)}$ is the estimation of ability in an exam as shown Figure 1, although it differs from the “true” estimation. The superscript “^*^” denotes the aberrant response.

**Figure 2:**
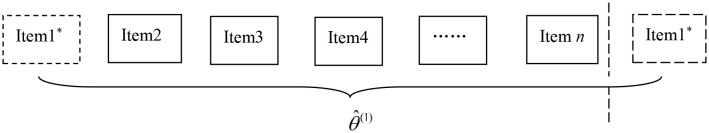
Estimation of ability ${\hat{\theta }}^{\left(1\right)}$ by MLE in aberrant responses.

Then we can resample the responses using the bootstrap method. If we select the first item (i.e., the aberrant response), and place it into the whole responses as shown in Figure 2, *n*+1 responses can be obtained. The estimation of ability, ${\hat{\theta }}^{\left(1\right)}$ is obtained using MLE. As the ratio of aberrant responses to the whole responses becomes larger, intuitively, ${\hat{\theta }}^{\left(1\right)}$ may be farther from “true” ability estimation than ${\hat{\theta }}^{\left(0\right)}$, and the absolute bias of ${\hat{\theta }}^{\left(1\right)}$ is larger than the absolute bias of ${\hat{\theta }}^{\left(0\right)}$.

**Figure 3:**
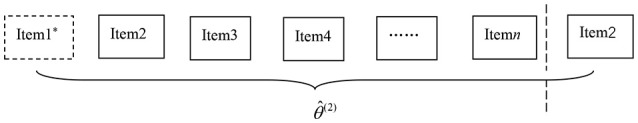
Estimation of ability ${\hat{\theta }}^{\left(2\right)}$ by MLE in aberrant responses.

However, if we resample the second item (i.e., a non-aberrant response) as shown in Figure 3, there are also *n*+1 responses, containing only one aberrant response (i.e., item 1^*^). The estimation, ${\hat{\theta }}^{\left(2\right)}$, is obtained by MLE. As the ratio of the aberrant responses to the whole responses is reduced, the absolute bias of ${\hat{\theta }}^{\left(2\right)}$ may become smaller than ${\hat{\theta }}^{\left(0\right)}$.

In order to determine the above ideas, the bias formula under aberrant responses needs to be determined. Lord (1981) derived the formula of bias that had been widely used to judge the accuracy of estimation under IRT framework. However, Lord's formula based on the ideal state did not consider aberrant responses.

Following Lord's idea, this paper aims (1) to present the generalized formula of statistical bias in the maximum likelihood estimation with or without aberrant responses, (2) to present, test and illustrate the utility of the proposed evaluation criterion which depends on the statistical bias.

## Statistical Bias of Aberrant Responses

In conventional IRT models, the probability of a correct item response depends on the characteristics of items and respondents. For instance, in the popular two-parameter logistic (2PL) model (Birnbaum, 1968), the probability of a correct response is in the form of

(1) $$\begin{array}{r} P_{r}\left(u_{i}=1|\theta ,a_{i},b_{i}\right)=\frac{\exp\left(a_{i}\left(\theta -b_{i}\right)\right)}{1+\exp\left(a_{i}\left(\theta -b_{i}\right)\right)}\\ =\frac{1}{1+\exp\left(-a_{i}\left(\theta -b_{i}\right)\right)} \end{array}$$

where θ is the ability of the individual, *a*_*i*_ is the item discrimination parameter, and *b*_*i*_ is the item difficulty parameter with *i* = 1,…, *n*, indexing items. The items are scored dichotomously, *u*_*i*_ = 1 for a correct response and *u*_*i*_ = 0 for an incorrect response. The examinee subscript is omitted to simplify notation throughout the paper.

The probability of a correct non-aberrant response can be expressed as

(2) $$\begin{array}{r} P_{i}=P_{i}\left(\theta \right)=\mathrm{Pr}\left(u_{i}=1|\theta ,a_{i},b_{i}\right) \end{array}$$

Define *Q*_*i*_(θ) = 1−*P*_*i*_(θ), *Q*_*i*_ = 1−*P*_*i*_ as the probability of an incorrect response to item *i*. The likelihood function is given by

(3) $$\begin{array}{r} L\left(\theta \right)=\overset{n}{\underset{i=1}{\prod }}{P_{i}}^{u_{i}}{Q_{i}}^{1-u_{i}} \end{array}$$

Then the log likelihood function is

(4) $$\begin{array}{r} l\left(\theta \right)=\log L\left(\theta \right)=\overset{n}{\underset{i=1}{\sum }}\left(u_{i}\log P_{i}\left(\theta \right)+\left(1-u_{i}\right)\log Q_{i}\left(\theta \right)\right) \end{array}$$

The maximum likelihood estimator (MLE) of ability, $\hat{\theta }$, is obtained by solving the non-linear equation as follows:

(5) $$\begin{array}{r} \frac{dl\left(\theta \right)}{d\theta }=\overset{n}{\underset{i}{\sum }}\frac{dl_{i}\left(\theta \right)}{d\theta }=\overset{n}{\underset{i=1}{\sum }}\left(u_{i}-{\hat{P}}_{i}\right)a_{i}=0 \end{array}$$

where ${\hat{P}}_{i}=P_{i}\left(\hat{\theta }\right)$, and *l*_*i*_(θ) = *u*_*i*_log*P*_*i*_(θ) + (1−*u*_*i*_)log*Q*_*i*_(θ).

Rewrite (5) as

(6) $$\begin{array}{r} {\hat{L}}_{1}\equiv \overset{n}{\underset{i}{\sum }}{\hat{\Gamma }}_{1i}=0 \end{array}$$

where by definition

(7) $$\begin{array}{r} \Gamma_{si}=\frac{d^{s}}{d\theta^{s}}\log{P_{i}}^{u_{i}}{Q_{i}}^{1-u_{i}} \end{array}$$

Thus, ${\hat{L}}_{1}$ considered as a function of $\hat{\theta }$ can be expanded formally in powers of $x\equiv \hat{\theta }-\theta$ , as follows:

(8) $$\begin{array}{r} {\hat{L}}_{1}\equiv \overset{n}{\underset{i}{\sum }}\Gamma_{1i}+\left(\hat{\theta }-\theta \right)\overset{n}{\underset{i}{\sum }}\Gamma_{2i}+\frac{1}{2}{\left(\hat{\theta }-\theta \right)}^{2}\overset{n}{\underset{i}{\sum }}\Gamma_{3i}+\cdots \cdots \end{array}$$

Let $\Gamma_{s}=\overset{n}{\underset{i}{\sum }}\Gamma_{si}$. Rather than proving the convergence of the power series, let us use a closed form that is always valid:

(9) $$\begin{array}{r} {\hat{L}}_{1}=\Gamma_{1}+x\Gamma_{2}+\frac{1}{2}x^{2}\Gamma_{3}+\frac{1}{6}x^{3}\Gamma_{4}+\frac{1}{24}x^{4}\Gamma_{5} \end{array}$$

By defining

(10) $$\begin{array}{rcl} \gamma_{si} & \equiv & E\Gamma_{si}, \end{array}$$

(11) $$\begin{array}{rcl} \varepsilon_{si} & \equiv & \Gamma_{si}-E\Gamma_{si} \end{array}$$

(12) $$\begin{array}{rcl} \gamma_{s} & \equiv & \frac{1}{n}\overset{n}{\underset{i}{\sum }}\gamma_{si}, \end{array}$$

(13) $$\begin{array}{rcl} \varepsilon_{s} & \equiv & \frac{1}{n}\overset{n}{\underset{i}{\sum }}\varepsilon_{si}. \end{array}$$

We can obtain

(14) $$\begin{array}{r} n\Gamma_{s}=\varepsilon_{s}-\gamma_{s}. \end{array}$$

When the examinee has an aberrant response on item *i*, denote the aberrant response as ${u_{i}}^{*}$ and the probability of a correct response as *P*${}_{i}^{*}$. Because $Eu_{i}=P_{i},E{u_{i}}^{*}={P_{i}}^{*},$ when the response is aberrant, we find that

(15) $$\begin{array}{rcl} \Gamma_{1i} & = & a_{i}\left(u_{i}^{*}-P_{i}\right), \end{array}$$

(16) $$\begin{array}{rcl} \Gamma_{2i} & = & -{a_{i}}^{2}P_{i}Q_{i}, \end{array}$$

(17) $$\begin{array}{rcl} \Gamma_{3i} & = & -{a_{i}}^{3}P_{i}Q_{i}\left(1-2P_{i}\right), \end{array}$$

(18) $$\begin{array}{rcl} \gamma_{1i} & = & a_{i}\left({P_{i}}^{*}-P_{i}\right), \end{array}$$

(19) $$\begin{array}{rcl} \gamma_{2i} & = & -{a_{i}}^{2}P_{i}Q_{i}, \end{array}$$

(20) $$\begin{array}{rcl} \gamma_{3i} & = & -{a_{i}}^{3}P_{i}Q_{i}\left(1-2P_{i}\right), \end{array}$$

(21) $$\begin{array}{rcl} \varepsilon_{1i} & = & a_{i}\left({u_{i}}^{*}-P_{i}^{*}\right), \end{array}$$

(22) $$\begin{array}{rcl} \varepsilon_{2i} & = & \varepsilon_{3i}=0. \end{array}$$

Then, the Fisher information is

(23) $$\begin{array}{r} I\equiv -E\frac{dL_{1}}{d\theta }=-n\gamma_{2}. \end{array}$$

Set (9) equal to zero, then it can be rewritten in terms of

(24) $$\begin{array}{r} -\left(\varepsilon_{1}+\gamma_{1}\right)=x\left(\gamma_{2}+\varepsilon_{2}\right)+\frac{1}{2}x^{2}\left(\gamma_{3}+\varepsilon_{3}\right)\\ +\frac{1}{6}x^{3}\left(\gamma_{4}+\varepsilon_{4}\right)+\frac{1}{24}x^{4}\left(\gamma_{5}+\varepsilon_{5}\right). \end{array}$$

Take the expectation of (24) to obtain a closed form

(25) $$\begin{array}{r} Bias\left(\hat{\theta }\right)=Ex=-\frac{1}{\gamma_{2}^{}}\left(E\varepsilon_{1}+E\gamma_{1}+Ex\varepsilon_{2}+\frac{1}{2}\gamma_{3}Ex^{2}\right), \end{array}$$

where *Ex*^*r*^(r = 1, 2, ⋯) is of order *n*^−*r*/2^ Thus, *Ex* is of order *n*^−1/2^. $Ex^{r}\varepsilon_{i}^{t}$ is of order *n*^−(*r*+*t*)/2^,where *r, t* = 1, 2, … (Lord, 1981)

Using (21) and (22)

(26) $$\begin{array}{r} E_{1}\varepsilon_{r}=0.\;r=1,2,\cdots \end{array}$$

Square (24) and take expectation, because of local independence, we can obtain

(27) $$\begin{array}{r} \begin{array}{cc} E_{1}x^{2} & =\frac{1}{\gamma_{2}^{2}}\left(E_{1}\varepsilon_{1}^{2}+E_{1}\gamma_{1}^{2}+2\gamma_{1}E_{1}\varepsilon_{1}\right)\\ =\frac{n^{2}}{I^{2}}\left(\frac{1}{n^{2}}E_{1}\overset{\text{n}}{\underset{i=1}{\sum }}{\left(a_{i}^{}\left(u_{i}^{*}-P_{i}^{*}\right)\right)}^{2}+\frac{1}{n^{2}}E_{1}\overset{n}{\underset{i=1}{\sum }}{\left(a_{i}^{}\left(P_{i}^{*}-P_{i}^{}\right)\right)}^{2}\right)\;\\ =\frac{1}{I^{2}}\left(\overset{n}{\underset{i=1}{\sum }}\left(a_{i}^{2}\left(P_{i}^{*}+P_{i}^{2}-2P_{i}^{*}P_{i}\right)\right)\right) \end{array} \end{array}$$

Take (26, 27) and (18) into (25) to obtain the formula of aberrant response bias

(28) $$\begin{array}{r} Bias\left(\hat{\theta }\right)=\frac{1}{I}G_{\left(n\right)}-\frac{1}{2I^{3}}H_{\left(n\right)}=\frac{2I^{2}G_{\left(n\right)}-H_{\left(n\right)}}{2I^{3}}, \end{array}$$

where

(29) $$\begin{array}{r} G_{\left(n\right)}=\overset{n}{\underset{i=1}{\sum }}a_{i}\left(P_{i}^{*}-P_{i}\right), \end{array}$$

(30) $$\begin{array}{r} H_{\left(n\right)}=\overset{n}{\underset{i=1}{\sum }}a_{i}{P_{i}}^{\prime \prime }\overset{n}{\underset{i=1}{\sum }}a_{i}^{2}\left(P_{i}^{*}+P_{i}^{2}-2P_{i}^{*}P_{i}\right). \end{array}$$

When the response is non-aberrant (i.e., $P_{i}^{*}=P_{i}$), and G_(n)_ = 0, the aberrant bias degenerates to the normal bias (Warm, 1989), that is

(31) $$\begin{array}{r} Bias\left(\hat{\theta }\right)=-\frac{\overset{}{\underset{i}{\sum }}a_{i}^{3}P_{i}Q_{i}\left(1-2P_{i}\right)}{2I^{2}}=-\frac{J}{2I^{2}}, \end{array}$$

where

$$\begin{array}{l} J=\overset{}{\underset{i}{\sum }}\frac{{P^{\prime }}_{i}{P^{\prime \prime }}_{i}}{P_{i}Q_{i}}. \end{array}$$

For an *n*-item test, when *s* aberrant and *n-s* non-aberrant responses are present, the absolute bias is

(32) $$\begin{array}{r} |Bias\left(\hat{\theta }\right)|=|\frac{-H_{\left(s\right)}+2I^{2}G_{\left(n-s\right)}-H_{\left(n-s\right)}}{2I^{3}}|. \end{array}$$

Formula (32) shows how non-aberrant and aberrant responses affect estimation ability. Based on the above formula, in the following section, a new detecting method is proposed.

## Aberrant Absolute Bias

Obviously, when the aberrant response occurs, the estimation of ability will drift off the “true” ability estimate and the true ability. Accordingly, when to resample a response from the dataset and re-estimate the ability, if the selected response is normal, the difference between the ability estimations can be negligible, however, selecting an aberrant response will increase the difference between the ability estimations. Hence, we can say that if the difference between two ability estimates locates in a pre-defined range, the examinee may have aberrant responses with a high probability. According to the idea above, the accuracy of the estimation for aberrant responses is affected by the ratio of aberrant responses to the whole responses.

### Example

Assuming *P*_1_^*^ = 0.25 is the probability of correct aberrant response on the first item, and *P*_*i*_ = 0.2 for *i* = 1, 2, 3…, *n* are the probabilities of correct non-aberrant responses. If we select the *n*th item (i.e., a normal response) and put it into the whole responses, we can obtain *n*+1 responses. Denote *P*_*n*__+1_ = 0.2, which means the response of the first item is aberrant, and others (2 to *n*+1) are not. According to Equation (28), we can obtain

$$\begin{array}{l} {\left|Bias\text{(}\hat{\theta }\text{)}\right|}_{\left(n+1\right)}=|\frac{2I^{2}G_{\left(n+1\right)}-H_{\left(n+1\right)}}{2I^{3}}|\\ =|\frac{2{\left(\overset{n+1}{\underset{i=1}{\sum }}I_{i}\right)}^{2}\left(\overset{n+1}{\underset{i=1}{\sum }}a_{i}\left(P_{i}^{*}-P_{i}\right)\right)-\overset{n+1}{\underset{i=1}{\sum }}a_{i}{{P^{\prime \prime }}_{i}}_{i}\overset{n+1}{\underset{i=1}{\sum }}a_{i}^{2}\left(P_{i}^{*}+{P_{i}}^{2}-2P_{i}^{*}P_{i}^{}\right)}{2{\left(\overset{n+1}{\underset{i=1}{\sum }}I_{i}\right)}^{3}}|\\ <|\frac{2{\left(\overset{n}{\underset{i=1}{\sum }}I_{i}\right)}^{2}G_{\left(n\right)}-H_{\left(n\right)}}{2{\left(\overset{n}{\underset{i=1}{\sum }}I_{i}\right)}^{3}}|={\left|Bias\text{(}\hat{\theta }\text{)}\right|}_{\left(n\right)} \end{array}$$

where ${\left|Bias\left(\hat{\theta }\right)\right|}_{\left(n\right)}$ is the absolute bias of the original *n* items, and ${\left|Bias\left(\hat{\theta }\right)\right|}_{\left(n+1\right)}$ is the absolute bias after resample the non-aberrant response.

### Formulation of the New Evaluation Criterion

Based on the above ideas, resampling aberrant responses or non-aberrant responses will result in different ability estimates. Therefore, we propose a new evaluation criterion named the aberrant absolute bias (|ABIAS|), which can be summarized as follows,

(33) $$\begin{array}{r} \left|\text{ABIAS}|\equiv \frac{1}{n}\overset{n}{\underset{i=1}{\sum }}|\left({\hat{\theta }}_{}^{\left(i\right)}-{\hat{\theta }}_{}^{\left(0\right)}\right)\right| \end{array}$$

where ${\hat{\theta }}^{\left(0\right)}$ is the estimation of ability with response pattern **u**^**(0)**^ = (*u*_1_, *u*_2_, *u*_3_,…, *u*_*n*_) using MLE method, and ${\hat{\theta }}^{\left(i\right)},\;i=1,2,3\cdots n$, is the estimation of ability with response **u**^**(i)**^ = (*u*_1_, *u*_2_, *u*_3_,…, *u*_*n*_, *u*_*i*_) using MLE method. To alleviate any propagation of errors from item parameter calibration, |ABIAS| have to be used with restriction on item parameters, which are constrained to be known or pre-calibrated accurately. Throughout this paper, we assume the item parameters are known.

|ABIAS| describes the deviation of expanding one response (*u*_*i*_, *i* = 1, 2, 3…*n*) each time from the original responses. Selecting the first response data *u*_1_ from whole responses **u**^**(0)**^ to **u**^**(0)**^, then **u**^**(0)**^ turns to **u**^**(1)**^ = (*u*_1_, *u*_2_, *u*_3_,…, *u*_*n*_, *u*_1_). So an ability estimation ${{\hat{\theta }}_{}}^{\left(1\right)}$ can be obtained from the responses **u**^(1)^ by MLE method. Then we resample *u*_*i*_ (*i* = 2,…, *n*) from **u**^**(0)**^ in sequence, and repeat *n-*1 times. Then we can obtain ${{\hat{\theta }}_{}}^{\left(2\right)},{{\hat{\theta }}_{}}^{\left(3\right)},\cdots \cdots {{\hat{\theta }}_{}}^{\left(n\right)}$ from **u**^**(i)**^ = (*u*_1_, *u*_2_, *u*_3_,…, *u*_*n*_, *u*_*i*_) (*i* = 2, …, *n*). Note that each estimation process only base on *n*+1 data.

|ABIAS| provides a new method based on bootstrap to detect aberrant examinee roughly by migration of the “true” ability estimation. The calculation of |ABIAS| is based on MLE method. It will carry out *n*+1 MLE operations for a *n-*item test. As we known, the MLE is very fast. So |ABIAS| can be used for a quick pre-screening. For example, it could help determine whether aberrant responses exist in a computer-based test before using RTs methods.

### Judgment Process

The judgment process by |ABIAS| can be summarized in three steps.

#### Sign-Process

For a given test, as the item parameters are (assumed to be) known, drawing some abilities from U(-3, 3) and simulating the corresponding response data, then |ABIAS| of abilities from −3 to 3 can be calculated. We call this process sign-process (SP). Denote the |ABIAS| calculated in this step as |ABIAS|_SP_. The |ABIAS|_SP_ are based on the assumption of non-aberrant response, which are the benchmarks for our judgment for aberrant examinees.

#### Measure-Process

Estimating the abilities and calculating the |ABIAS| for each examinee. We call this step measure-process (MP), and denote the |ABIAS| calculated here as |ABIAS|_MP_. For each examinee, |ABIAS|_MP_ has only one value.

#### Compare-Process

Comparing |ABIAS|_MP_ to |ABIAS|_SP_. This process is called compare-process (CP). If |ABIAS|_MP_ falls into the range of |ABIAS|_SP_, the responses are determined to be non-aberrant. Otherwise, responses are aberrant.

The method based on |ABIAS| to determine whether aberrant responses exist is called the |ABIAS| method.

## Simulation Studies

A large number of studies had focused on aberrant responses. Mislevy and Bock (1982) recommended Tukey's bisquare weight function (Mosteller and Tukey, 1977) to handle aberrant responses, whereas Schuster and Yuan (2011) suggested Huber-type weight function to enhance estimation effect. All these studies used the same method to generate item parameters (Donoghue and Allen, 1993; Zwick et al., 1993; Penfield, 2003; Magis, 2014). Hence, to maintain consistency with their researches, all item parameters in simulation studies were same as those in Magis (2014), as shown in Table 1.

**Table 1:** Item discrimination *a*_*i*_ and difficulty parameters *b*_i_ in Magis (2014).

**Item**	***a_***i***_***	***b_***i***_***	**Item**	***a_***i***_***	***b_***i***_***	**Item**	***a_***i***_***	***b_***i***_***
1	0.799	−1.961	21	1.317	4.271	41	1.049	−0.665
2	1.192	0.561	22	0.182	−2.557	42	0.848	−3.889
3	1.100	3.106	23	1.115	3.125	43	0.901	−1.711
4	0.910	−2.243	24	1.081	3.775	44	1.129	3.732
5	1.032	−0.113	25	0.842	−0.199	45	0.992	1.162
6	0.950	0.187	26	0.915	−4.726	46	1.416	4.171
7	0.915	1.226	27	0.994	0.892	47	1.046	−0.522
8	0.942	−0.524	28	0.924	−1.219	48	1.160	0.008
9	1.214	3.972	29	1.046	1.546	49	0.951	−0.408
10	0.920	−0.362	30	1.059	0.615	50	0.869	−2.428
11	0.968	0.747	31	1.215	1.627	51	0.853	−1.772
12	1.126	1.993	32	1.175	0.796	52	1.192	4.104
13	0.953	−0.798	33	1.009	2.038	53	0.984	−1.079
14	0.938	−2.036	34	0.876	−0.696	54	1.254	2.677
15	1.178	3.555	35	0.826	−1.694	55	0.947	−2.040
16	0.749	−7.403	36	0.857	−1.308	56	0.861	−3.884
17	1.203	1.878	37	0.990	−3.263	57	1.068	−0.529
18	1.066	0.144	38	0.919	−1.802	58	1.195	1.978
19	1.195	2.123	39	0.888	−1.196	59	1.123	2.286
20	0.933	0.730	40	0.932	−0.549	60	1.102	3.266

To evaluate the performance of |ABIAS| for 2PL models, two simulation studies were conducted. The manipulated factors, which were same in both studies, included 3 levels of test length (20, 40, and 60) which represented short, moderate, and long tests, and 7 levels of ability (from −3 to 3 with step 1). As the detection procedure for each examinee was independent, in this section, we focused solely on one examinee. More precisely, tests of 20 (40) items were generated by using the first 20 (40) item parameters of Table 1.

Simulation study 1 was based on the random aberrant process in three scenarios to evaluate the performance of the proposed |ABIAS| method. For the random aberrant process, if the response *u*_*i*_ was 1, then it will be changed to 0, otherwise, it will be changed to 1. The random aberrant process do not focus on the source of aberrant responses. An additional simulation check to compare with *l*_*z*_ was provided in Appendix (Supplementary Material).

Simulation study 2 was based on the aberrant guessing process used by Schuster and Yuan (2011) and Magis (2014). The aberrant guessing process assumed that one will answer the *i*th item aberrantly if the probability of the correct response is less than *P*^#^, where *P*^#^ was a pre-defined cut-off value. In other words, the examinee will guess randomly when the probability of answering the item correctly was less than *P*^#^. Thus, any item response with correct response probability less than *P*^#^ was replaced by an aberrant response with probability P^*^. P^*^ was the pre-defined probability of the correct aberrant response in aberrant guessing process.

### Step SP

In Table 2, we generated 13 intervals of abilities θ from −3 to 3 with step 0.5. Response data were generated from the 2PL model with item parameters in Table 1 under the non-aberrant assumption. MLE method was used to estimate abilities. Because of the biased property of the MLE method, considering the range of ability was more reasonable than considering the ability point. Five hundred replications were done.

**Table 2:** Summary of |ABIAS|_SP_.

**Ability range**	***N*** **=** **20**						***N*** **=** **40**						***N*** **=** **60**					
	**MAX**	**MIN**	**MEAN**	**68%-P**	**95%-P**	**99%-P**	**MAX**	**MIN**	**MEAN**	**68%-P**	**95%-P**	**99%-P**	**MAX**	**MIN**	**MEAN**	**68%-P**	**95%-P**	**99%-P**
[2.5,3]	0.176	0.060	0.088	0.096	0.123	0.154	0.071	0.035	0.050	0.053	0.062	0.068	0.045	0.025	0.034	0.035	0.041	0.043
[2,2.5]	0.161	0.060	0.089	0.096	0.123	0.154	0.072	0.038	0.050	0.053	0.063	0.067	0.045	0.027	0.033	0.035	0.039	0.042
[1.5,2]	0.157	0.060	0.091	0.098	0.117	0.128	0.072	0.038	0.050	0.052	0.060	0.065	0.045	0.026	0.033	0.034	0.039	0.042
[1,1.5]	0.157	0.067	0.091	0.097	0.117	0.128	0.071	0.038	0.050	0.052	0.060	0.064	0.043	0.024	0.033	0.034	0.038	0.040
[0.5,1]	0.157	0.067	0.090	0.095	0.116	0.126	0.071	0.038	0.048	0.051	0.058	0.062	0.043	0.024	0.032	0.033	0.037	0.040
[0,0.5]	0.150	0.064	0.090	0.095	0.112	0.124	0.067	0.036	0.047	0.049	0.056	0.060	0.044	0.024	0.032	0.032	0.037	0.040
[−0.5,0]	0.138	0.064	0.089	0.093	0.111	0.124	0.065	0.036	0.047	0.049	0.055	0.060	0.039	0.023	0.031	0.032	0.036	0.039
[−1,−0.5]	0.134	0.058	0.086	0.091	0.108	0.119	0.065	0.034	0.046	0.048	0.055	0.060	0.040	0.022	0.030	0.032	0.035	0.037
[−1.5,−1]	0.134	0.058	0.086	0.091	0.108	0.119	0.065	0.033	0.046	0.044	0.054	0.058	0.041	0.023	0.029	0.030	0.034	0.036
[−2,−1.5]	0.132	0.058	0.082	0.087	0.106	0.118	0.064	0.033	0.043	0.045	0.052	0.055	0.040	0.021	0.029	0.030	0.034	0.036
[−2.5,−2]	0.133	0.058	0.082	0.087	0.106	0.116	0.061	0.032	0.043	0.045	0.052	0.055	0.037	0.021	0.028	0.029	0.032	0.034
[−3,−2.5]	0.127	0.058	0.078	0.082	0.100	0.116	0.064	0.030	0.042	0.044	0.051	0.055	0.036	0.020	0.028	0.029	0.032	0.035
Mean	0.146	0.061	0.086	0.092	0.112	0.127	0.067	0.035	0.046	0.048	0.056	0.060	0.041	0.023	0.031	0.032	0.036	0.038
SD	0.015	0.003	0.004	0.004	0.007	0.013	0.003	0.002	0.002	0.003	0.004	0.004	0.003	0.002	0.002	0.002	0.002	0.002

*Mean denotes the corresponding sample mean and SD is the sample standard deviation*.

Table 2 shows the |ABIAS|_SP_ in 3 levels of test length. MAX is the maximum value of 500 times, MIN is the minimum value, and MEAN is the mean value of 500 times. 68%-P is the value of the position of 68% in ascending order, and so are 95%-P and 99%-P. They correspond to the three standard errors of standard normal distribution. The value of |ABIAS|_SP_ in 60-item test was smaller than that in 20- and 40-item tests. That is because the greater the item length, the higher the accuracy of ability estimation. In Table 2, the empirical standard deviations of MIN, MEAN and 68%-P were all smaller than those of MAX, 95%-P and 99%-P, that is, the stabilities of MIN, MEAN and 68%-P are better than other indices.

Empirically, |ABIAS|_SP_ in an interval was monotonous, so we only calculated the two endpoints of each ability interval and take the smaller value as the criterion. Because the probability that the estimated value locates in the endpoints was close to 0, the intervals were set to be closed.

There are two plans in CP. If we want a more accuracy judgment, we can choose the |ABIAS|_SP_ value (such as 68%-P, 68%-m-P, 95%-P, 95%-m-P) in the ability ranges. If we want a more quickly judgment, we can choose the mean value of indices (such as mean of 68%-P). For example, if one's |ABIAS|_MP_ is smaller than the 68%-P of |ABIAS|_SP_, it can be marked as non-aberrant. What's more, Table 2 gives us different choices. If we want to have higher accuracy in detecting aberrant responses, we can use 95%-P and 95%-m-P. If we want to retain all the possible non-aberrant examinees, we can use 68%-P or 68%-m-P. This is a trade-off between detection of aberrant examinees and retention of normal examinees.

About 0.013 second of CPU time for each replication was required on a 1.60 GHz desktop using MATLAB 2016a.

### Simulation Study 1

This simulation study was conducted to measure the |ABIAS| method in the random aberrant process. Under each condition, 3 levels of aberrant proportion (5, 15, and 25%) were considered, and the aberrant responses were selected randomly with the aberrant proportion. Across all the conditions, 500 replications were conducted. The results from the MP step were summarized in Tables 3–5.

**Table 3:** Summary of MP and CP in random aberrant process in scenario 1.

**Aberrant response**	**θ**	**$$$$**	**|ABIAS|**_****MP****_			**Correct detection frequency**			
			**MAX**	**MIN**	**MEAN**	**LT-68%**	**LT-m-68%**	**LT-95%**	**LT-m-95%**
1	3	2.658	0.182	0.060	0.107	393	411	94	185
	2	1.791	0.183	0.060	0.105	326	269	124	160
	1	0.874	0.154	0.067	0.100	312	352	97	135
	0	−0.010	0.157	0.066	0.096	296	315	72	67
	−1	−0.972	0.131	0.064	0.092	276	266	69	42
	−2	−1.918	0.142	0.058	0.090	287	216	72	42
	−3	−2.758	0.133	0.058	0.089	310	175	82	29
3	3	1.637	0.185	0.060	0.128	453	464	328	360
	2	1.195	0.190	0.067	0.120	439	466	252	298
	1	0.571	0.180	0.070	0.111	429	477	205	257
	0	−0.209	0.155	0.070	0.107	407	414	183	176
	−1	−0.953	0.157	0.064	0.102	415	406	195	151
	−2	−1.638	0.139	0.063	0.100	412	360	196	120
	−3	−2.256	0.154	0.058	0.101	413	361	195	141
5	3	1.087	0.202	0.079	0.137	484	494	447	416
	2	0.723	0.191	0.076	0.127	473	481	417	369
	1	0.276	0.182	0.070	0.120	448	461	365	315
	0	−0.251	0.179	0.070	0.114	447	455	321	283
	−1	−0.852	0.162	0.071	0.111	449	442	297	265
	−2	−1.476	0.166	0.067	0.109	437	439	268	241
	−3	−1.963	0.161	0.069	0.112	463	432	311	262

**Table 4:** Summary of MP and CP in random aberrant process in scenario 2.

**Aberrant response**	**θ**	**$$$$$**	**|ABIAS|**_****MP****_			**Correct detection frequency**			
			**MAX**	**MIN**	**MEAN**	**LT-68%**	**LT-m-68%**	**LT-95%**	**LT-m-95%**
2	3	2.345	0.088	0.043	0.061	380	463	124	305
	2	1.583	0.089	0.039	0.058	362	452	141	259
	1	0.796	0.076	0.040	0.055	342	428	138	190
	0	−0.117	0.068	0.038	0.053	331	360	116	98
	−1	−0.930	0.071	0.035	0.051	316	316	81	60
	−2	−1.784	0.066	0.034	0.049	358	246	120	44
	−3	−2.581	0.066	0.033	0.049	363	208	112	36
6	3	1.394	0.098	0.048	0.073	494	499	432	473
	2	0.916	0.096	0.048	0.069	481	495	416	442
	1	0.444	0.089	0.043	0.064	480	490	393	393
	0	−0.174	0.081	0.039	0.061	462	473	324	303
	−1	−0.784	0.079	0.043	0.059	444	444	250	219
	−2	−1.433	0.083	0.040	0.059	484	441	278	231
	−3	−1.967	0.079	0.038	0.060	486	469	392	228
10	3	1.307	0.101	0.052	0.074	500	500	482	498
	2	0.889	0.097	0.048	0.070	499	500	463	476
	1	0.410	0.089	0.047	0.066	496	497	449	449
	0	−0.189	0.085	0.044	0.062	489	493	426	409
	−1	−0.803	0.081	0.044	0.060	484	484	385	365
	−2	−1.381	0.081	0.043	0.060	499	484	395	347
	−3	−1.895	0.083	0.033	0.061	498	491	453	375

**Table 5:** Summary of MP and CP in random aberrant process in scenario 3.

**Aberrant response**	**θ**	**$$$$**	**|ABIAS|**_****MP****_			**Correct detection frequency**			
			**MAX**	**MIN**	**MEAN**	**LT-68%**	**LT-m-68%**	**LT-95%**	**LT-m-95%**
3	3	2.486	0.055	0.031	0.039	464	495	270	421
	2	1.653	0.048	0.030	0.038	419	471	186	341
	1	0.794	0.047	0.029	0.036	420	448	202	257
	0	−0.178	0.049	0.031	0.038	390	390	167	167
	−1	−0.796	0.044	0.027	0.035	316	316	155	115
	−2	−1.671	0.046	0.028	0.035	368	251	143	68
	−3	−2.468	0.048	0.028	0.036	391	206	206	38
9	3	1.836	0.059	0.031	0.045	499	500	466	497
	2	1.261	0.058	0.031	0.043	494	498	463	486
	1	0.617	0.053	0.031	0.041	489	493	425	455
	0	−0.093	0.048	0.028	0.039	494	494	404	404
	−1	−0.836	0.053	0.027	0.038	472	472	381	344
	−2	−1.594	0.050	0.024	0.037	494	467	418	330
	−3	−2.243	0.047	0.026	0.037	498	465	465	338
15	3	1.321	0.067	0.033	0.050	500	500	498	500
	2	0.918	0.060	0.034	0.047	500	500	498	496
	1	0.448	0.057	0.035	0.045	500	500	482	491
	0	−0.183	0.054	0.029	0.043	499	499	480	480
	−1	−0.762	0.052	0.034	0.043	499	499	482	465
	−2	−1.228	0.055	0.034	0.044	499	497	481	462
	−3	−1.692	0.052	0.038	0.045	499	498	491	466

In Tables 3–5, $\hat{\theta }$ was the mean value of ability estimations in 500 replications. LT-68% was the number of |ABIAS|_MP_ larger than 68% *P*-values in SP. LT-m-68% was the number of |ABIAS|_MP_ larger than mean of 68% *P*-values in SP. LT-95% was the number of |ABIAS|_MP_ larger than 95% *P*-values in SP. LT-m-95% was the number of |ABIAS|_MP_ larger than mean of 95% *P*-values in SP.

Tables 3–5 indicate that the more aberrant responses, the more effective of the |ABIAS| method. Using LT-68% or LT-m-68% are better than using LT-95% or LT-m-95%. And there is few differences between LT-68% and LT-m-68%. Specifically speaking, it appears that it is better to use LT-m-68% when the estimation of ability is positive, and it is better to use LT-68% when the estimation of ability is negative. The worst case is in scenario 1, when there is only 1 random aberrant response in 20 items, the accuracy is about 40%, in other cases, the accuracy is more than 80% when we use LT-68% and LT-m-68% as the criteria in practical applications.

The MAX, MIN, and MEAN values in Tables 3–5 are all larger than those in Table 2. These observations indicate that the more aberrant responses in a test, that is, the larger the aberrant proportion, the better the performance of the |ABIAS| method in detecting aberrant responses. When the aberrant proportion is 25%, the |ABIAS| method can almost detect the existence of all aberrant examinees correctly for all replications. The accuracy of judgment increase as the absolute ability of the examinee decreases. This is because when the ability of the examinee is close to 0, correct response probability and incorrect response probability is close in many items. Hence, it will be very difficult to determine whether an aberrant response exists.

### Simulation Study 2

This simulation study was designed to measure the |ABIAS| method in aberrant guessing process. The aberrant guessing process was used by Schuster and Yuan (2011) and David Magis (2014). In each scenario, test with four-choice and five-choice items were considered. That meant, the probability of correct aberrant response, P^*^, was 0.25 or 0.2 for a four-choice item or a five-choice item. In this simulation study, the probability of pre-defined cut-off value, *P*^#^, was set to 0.1. Hence, the correct response probability on one item, which was less than *P*^#^ (i.e., 0.1), will be replaced by P^*^ (i.e., 0.25 for four-choice item or 0.2 for five-choice item), and denoted the “updated” response on this item as the aberrant response.

Tables 6–8 show that the values of LT-68%, LT-m-68% LT-95%, LT-m-95% are larger in negative ability than these in positive ability. Because higher ability would lead to more success probabilities which is larger than *P*^#^. In fact, lower ability would likely result in aberrant responses (Schuster and Yuan, 2011; Magis, 2014). The accuracy of simulation study 2 is lower than that of study 1, because even when *P*_*i*_ is smaller than *P*^#^ and replaced by P^*^, the difference between them was still small (0.25–0.1 = 0.15, and 0.2–0.1 = 0.1). Thus, response *u*_*i*_ may not change. Nevertheless, these findings still indicate that the |ABIAS| method is effective in the aberrant guessing process. Although success is not guaranteed every time, it can also guarantee at least 50% accuracy by using LT-68% when P^*^ is only 0.1 higher than *P*^#^. If the ability is smaller than −2, accuracy will almost be larger than 80%, making it feasible as a rough screening method.

**Table 6:** Summary of MP and CP in aberrant guessing process in scenario 1.

P^*^	**θ**	**$$$$**	**|ABIAS|**_****MP****_			**Correct detection frequency**			
			**MAX**	**MIN**	**MEAN**	**LT-68%**	**LT-m-68%**	**LT-95%**	**LT-m-95%**
0.25	3	3.707	0.183	0.060	0.099	224	253	96	114
	2	2.302	0.186	0.060	0.101	247	290	106	133
	1	1.161	0.176	0.067	0.099	233	292	77	110
	0	0.131	0.147	0.064	0.096	257	298	86	86
	−1	−0.756	0.148	0.064	0.098	330	319	143	108
	−2	−1.635	0.166	0.062	0.100	411	351	196	143
	−3	−2.188	0.169	0.058	0.110	437	411	291	247
0.2	3	3.630	0.180	0.060	0.097	215	238	95	110
	2	2.269	0.182	0.060	0.099	240	285	93	121
	1	1.146	0.174	0.067	0.095	223	274	80	98
	0	0.108	0.151	0.066	0.094	250	286	78	78
	−1	−0.781	0.145	0.064	0.096	303	292	116	98
	−2	−1.706	0.171	0.058	0.097	349	312	154	120
	−3	−2.380	0.172	0.058	0.105	409	378	240	188

**Table 7:** Summary of MP and CP in aberrant guessing process in scenario 2.

P^*^	**θ**	**$$$$$**	**|ABIAS|**_****MP****_			**Correct detection frequency**			
			**MAX**	**MIN**	**MEAN**	**LT-68%**	**LT-m-68%**	**LT-95%**	**LT-m-95%**
0.25	3	3.174	0.083	0.038	0.053	224	346	61	162
	2	2.128	0.085	0.037	0.053	248	388	50	161
	1	1.160	0.077	0.037	0.052	293	402	93	183
	0	0.179	0.077	0.038	0.052	314	345	134	134
	−1	−0.754	0.073	0.035	0.053	382	382	185	162
	−2	−1.445	0.083	0.030	0.056	481	442	299	241
	−3	−1.994	0.100	0.037	0.062	492	473	433	360
0.2	3	3.105	0.084	0.038	0.051	201	327	51	145
	2	2.150	0.079	0.038	0.053	228	367	46	171
	1	1.121	0.081	0.037	0.052	239	376	76	137
	0	0.118	0.069	0.034	0.051	324	346	122	122
	−1	0.068	0.073	0.034	0.051	306	311	110	110
	−2	−1.556	0.075	0.033	0.053	435	369	280	174
	−3	−2.214	0.083	0.037	0.058	470	444	379	286

**Table 8:** Summary of MP and CP in aberrant guessing process in scenario 3.

P^*^	*****θ*****	**$$$$$**	**|ABIAS|**_****MP****_			**Correct detection frequency**			
			**MAX**	**MIN**	**MEAN**	**LT-68%**	**LT-m-68%**	**LT-95%**	**LT-m-95%**
0.25	3	3.072	0.053	0.026	0.035	247	372	58	211
	2	2.101	0.047	0.026	0.035	266	408	95	208
	1	1.128	0.049	0.027	0.035	328	424	145	240
	0	0.167	0.050	0.025	0.035	406	404	179	230
	−1	−0.689	0.051	0.025	0.036	423	423	299	260
	−2	−1.365	0.056	0.026	0.039	496	478	442	493
	−3	−2.007	0.060	0.030	0.043	500	498	498	455
0.2	3	3.097	0.052	0.026	0.034	256	385	43	195
	2	2.070	0.048	0.026	0.035	259	402	78	204
	1	1.106	0.046	0.025	0.035	306	405	209	207
	0	0.149	0.044	0.026	0.034	358	358	116	164
	−1	−0.751	0.049	0.023	0.034	379	379	241	203
	−2	−1.479	0.049	0.026	0.037	485	449	386	331
	−3	−2.220	0.055	0.027	0.039	495	473	473	384

The simulation studies reflect the effectiveness of the |ABIAS| method in random guessing and aberrant guessing processes. In the same aberrant proportion (aberrant responses to the whole responses) or the same probability of the correct aberrant response, the longer the test, the better the screening effect. In the same test length, the larger the aberrant proportion, the batter the screening effect. In the two aberrant processes, ability levels are all an important factor to affect the performance. We recommend LT-m-68% and LT-m-95% as the criteria for positive ability estimations, and LT-68% and LT-95% for negative ability estimations.

## Application to Real Data

This example was based on a pilot study on a sample of 1,624 examinees under 170 items. The organization also flagged 41 examinees as possible cheaters from a variety of statistical analysis and an investigative process that brought in other information. The data sets were analyzed in several papers (Sinharay, 2016; Cizek and Wollack, 2017; Eckerly, 2017).

As the |ABISA| method was under the assumption that item parameters are known or pre-calibrated accurately. The item parameters were calibrated firstly by 1,583 non-aberrant examinees, and this process was called the Sign Step in simulation. And then the item parameters were used to analyze the 41 examinees who may have aberrant responses, and this is the Measure Step.

The *l*_*z*_ index is used as a baseline. The formulation of *l*_*z*_ is as follows,

(34) $$\begin{array}{r} l_{z}=\left(l\left(\theta \right)-E\left(l\left(\theta \right)\right)\right)/\sqrt{v\left(l\left(\theta \right)\right)}, \end{array}$$

(35) $$\begin{array}{r} v\left(l\left(\theta \right)\right)=\overset{n}{\underset{i=1}{\sum }}\left(P_{i}\left(\theta \right)Q_{i}\left(\theta \right)\right){\left(\ln\;\left(\frac{P_{i}\left(\theta \right)}{Q_{i}\left(\theta \right)}\right)\right)}^{2} \end{array}$$

Although *l*_*z*_ is not perfect (Molenaar and Hoijtink, 1990, 1996), but *l*_*z*_ is still the most popular parametric person-fit statistics. The summary of |ABIAS|_SP_ by 2PL models was as provided in Table 9.

**Table 9:** Summary of |ABIAS|_SP_ by 2PL model.

**Ability range**	**|ABIAS|**_****SP****_					
	**MAX**	**MIN**	**MEAN**	**68%-P**	**95%-P**	**99%-P**
[2.5,3]	0.029	0.018	0.022	0.022	0.024	0.026
[2,2.5]	0.029	0.018	0.021	0.021	0.023	0.024
[1.5,2]	0.024	0.017	0.020	0.020	0.022	0.022
[1,1.5]	0.022	0.016	0.019	0.019	0.021	0.021
[0.5,1]	0.021	0.016	0.018	0.018	0.019	0.020
[0,0.5]	0.019	0.015	0.017	0.017	0.018	0.019
[−0.5,0]	0.018	0.015	0.016	0.017	0.017	0.018
[−1,−0.5]	0.017	0.014	0.016	0.016	0.017	0.017
[−1.5,−1]	0.017	0.014	0.015	0.016	0.016	0.017
[−2,−1.5]	0.017	0.014	0.015	0.016	0.016	0.016
[−2.5,−2]	0.017	0.014	0.015	0.016	0.016	0.016
[−3,−2.5]	0.017	0.014	0.015	0.016	0.016	0.017
Mean	0.020	0.015	0.017	0.017	0.018	0.019
SD	0.004	0.001	0.002	0.002	0.002	0.003

Table 9 indicated the |ABIAS_|SP_ of the 2PL model. The SD of MIN and MEAN was small. The estimations and |ABIAS|_MP_ of the 41 examinees were provided in Table 10.

**Table 10:** Summary of estimation abilities and |ABIAS|_MP_ by 2PL model.

**#Examinee**	**$$$$**	**|ABIAS|_**MP**_**	**#examinee**	**$$$$**	**|ABIAS|_**MP**_**
1	−3.474	0.018	22	0.962	0.018
2	−2.894	0.017	23	0.702	0.018
3	−3.126	0.017	24	1.393	0.019
4	−1.626	0.015	25	0.375	0.019
5	−1.563	0.015	26	0.698	0.017
6	−1.299	0.016	27	0.666	0.019
7	−1.241	0.016	28	1.212	0.019
8	−0.937	0.016	29	0.596	0.019
9	−0.873	0.016	30	1.101	0.019
10	−0.897	0.017	31	−0.062	0.016
11	−0.891	0.016	32	−0.057	0.017
12	−0.601	0.016	33	0.244	0.017
13	−0.440	0.017	34	0.470	0.018
14	−0.171	0.017	35	0.002	0.018
15	0.163	0.018	36	0.675	0.018
16	0.096	0.017	37	0.910	0.019
17	0.282	0.017	38	0.494	0.019
18	−0.149	0.017	39	0.677	0.018
19	0.250	0.017	40	1.635	0.021
20	1.219	0.019	41	1.891	0.022
21	0.201	0.018		

Table 10 showed that using the |ABIAS| method, 15 aberrant examinees can be determined by 68%-P, 14 aberrant examinees by 68%-m-P, 6 aberrant examinees by 95%-P, and 12 aberrant examinees by 95%-m-P. Using the *l*_*z*_ index, 3 aberrant examinees can be identified by 2PL model. All the results by the |ABIAS| method covered the results by *l*_*z*_. It appears that the |ABIAS| method outperforms *l*_*z*_.

## Discussion and Conclusion

Aberrant responses often occurred in educational measurement. Most examinees can improve their scores by guessing when they did not know the answer, which may make it harder to obtain the “true” ability estimations. Hence, developing a simple and feasible screening method was necessary. At the very least, determining whether an examinee had aberrant responses in the test should be done. This was the main purpose of this article.

This paper followed the idea of Lord (1981) and provided a generalized formula of statistical bias in the maximum likelihood estimation with or without aberrant responses, which presented the relationship between bias and the probability of aberrant response. It was the first attempt to formulate the bias with aberrant responses, and the new bias was equivalent to the normal bias (Warm, 1989) when there were no aberrant responses in the test. The formula showed the estimation bias of aberrant responses consisted of two parts. One part came from non-aberrant responses, and the other came from aberrant responses. It was the basic of the |ABIAS| method.

In this paper, the |ABIAS| was proposed as a new indicator to identify aberrant responses according to the formula, which was fast and effective. Simulation studies showed that to a certain extent, the |ABIAS| method could judge whether an examinee had aberrant responses in a test in two different aberrant processes. The results indicated that in the random aberrant process the larger absolute ability, the better the detecting effect. In the aberrant guessing process, the smaller the ability, the better the detecting effect. Moreover, the larger the aberrant proportion, the higher the accuracy of detecting. The more items in the test, the better the detecting effect.

The new method does not rely on response times, which means that it can be used more widely. The paper-and-pencil tests can be screened by the new method and then the weight method can be used to obtain the robust estimation of examinee's ability with aberrant responses. Meanwhile, in computer-based tests, the new method can be used for screening firstly, and then the RTs can be used for the accurate search. This feature can save significant manpower and time.

In this article, the proposed detecting method is limited to unidimensional IRT models. However, as identified by Ackerman et al. (2003), many educational and psychological tests are inherently multidimensional. In multidimensional IRT (MIRT) models, the correlations between domains will affect the statistical biases of latent traits (Wang, 2015). In other words, the aberrant behavior on one item may affect the statistical biases of all the domains, rather than that of the corresponding domain. Therefore, future research should look into the application of the |ABIAS| method to detect aberrant responses under MIRT framework.

What's more, the new method could not identify which item is aberrant. In future research, we wish to construct a method based on |ABIAS| to determine which item the aberrant response occur on. This direction is a very interesting one and will have wider applications in computer-based testings.

## Author Contributions

Author Contributions ZZ completed the writing of the article. XZ provided key technical support. BJ provided original thoughts and article revisions.

### Conflict of Interest Statement

The authors declare that the research was conducted in the absence of any commercial or financial relationships that could be construed as a potential conflict of interest.
