# Supplementary material for: A Short Note on Aberrant Responses Bias in Item Response Theory
Source: Front Psychol. 2019 Jan 31;10:43. doi: 10.3389/fpsyg.2019.00043 (PMC6365413; doi:10.3389/fpsyg.2019.00043)
Supplement: Supplementary file 1 [file Data_Sheet_1.docx]

**Appendix. A simulation check to compare with** *l_z_*

In this additional study, only 60-item test was considered because *l_z_* did not perform well when test length was smaller than 50 (Meijer, 2001). 500 replications were done. Item parameters are the same as those in the ‘Simulation Studies’ section. Other design was the same as the ‘Simulation Studies’ section (i.e., number of aberrant responses and guessing processes). The results were provided in Tables A1 and A2.

**Table A1.** Summary of MP and CP in random aberrant process

| aberrant  response | *θ* | $\hat{\theta}$ | \|ABIAS\| | | | Correct detection Frequency | | | | |
| --- | --- | --- | --- | --- | --- | --- | --- | --- | --- | --- |
|  |  |  | MAX | MIN | MEAN | LT–  68% | LT–  m-68% | LT–  95% | LT–  m-95% | LT–*l_z_* |
| 3 | 3 | 2.486 | 0.055 | 0.031 | 0.039 | 464 | 495 | 270 | 421 | 256 |
|  | 2 | 1.653 | 0.048 | 0.030 | 0.038 | 419 | 471 | 186 | 341 | 216 |
|  | 1 | 0.794 | 0.047 | 0.029 | 0.036 | 420 | 448 | 202 | 257 | 183 |
|  | 0 | -0.178 | 0.049 | 0.031 | 0.038 | 390 | 390 | 167 | 167 | 148 |
|  | -1 | -0.796 | 0.044 | 0.027 | 0.035 | 316 | 316 | 155 | 115 | 135 |
|  | -2 | -1.671 | 0.046 | 0.028 | 0.035 | 368 | 251 | 143 | 68 | 169 |
|  | -3 | -2.468 | 0.048 | 0.028 | 0.036 | 391 | 206 | 206 | 38 | 229 |
| 9 | 3 | 1.836 | 0.059 | 0.031 | 0.045 | 499 | 500 | 466 | 497 | 467 |
|  | 2 | 1.261 | 0.058 | 0.031 | 0.043 | 494 | 498 | 463 | 486 | 456 |
|  | 1 | 0.617 | 0.053 | 0.031 | 0.041 | 489 | 493 | 425 | 455 | 418 |
|  | 0 | -0.093 | 0.048 | 0.028 | 0.039 | 494 | 494 | 404 | 404 | 398 |
|  | -1 | -0.836 | 0.053 | 0.027 | 0.038 | 472 | 472 | 381 | 344 | 394 |
|  | -2 | -1.594 | 0.050 | 0.024 | 0.037 | 494 | 467 | 418 | 330 | 414 |
|  | -3 | -2.243 | 0.047 | 0.026 | 0.037 | 498 | 465 | 465 | 338 | 471 |
| 15 | 3 | 1.321 | 0.067 | 0.033 | 0.050 | 500 | 500 | 498 | 500 | 496 |
|  | 2 | 0.918 | 0.060 | 0.034 | 0.047 | 500 | 500 | 498 | 496 | 494 |
|  | 1 | 0.448 | 0.057 | 0.035 | 0.045 | 500 | 500 | 482 | 491 | 482 |
|  | 0 | -0.183 | 0.054 | 0.029 | 0.043 | 499 | 499 | 480 | 480 | 483 |
|  | -1 | -0.762 | 0.052 | 0.034 | 0.043 | 499 | 499 | 482 | 465 | 479 |
|  | -2 | -1.228 | 0.055 | 0.034 | 0.044 | 499 | 497 | 481 | 462 | 490 |
|  | -3 | -1.692 | 0.052 | 0.038 | 0.045 | 499 | 498 | 491 | 466 | 495 |

**Table A2**. Summary of MP and CP in aberrant guessing process

| P*^*^* | *θ* | $\hat{\theta}$ | \|ABIAS\| | | | Correct detection Frequency | | | | |
| --- | --- | --- | --- | --- | --- | --- | --- | --- | --- | --- |
|  |  |  | MAX | MIN | MEAN | LT–  68% | LT–  m-68% | LT–  95% | LT–  m-95% | LT  –*l_z_* |
| 0.25 | 3 | 3.072 | 0.053 | 0.026 | 0.035 | 247 | 372 | 58 | 211 | 22 |
|  | 2 | 2.101 | 0.047 | 0.026 | 0.035 | 266 | 408 | 95 | 208 | 52 |
|  | 1 | 1.128 | 0.049 | 0.027 | 0.035 | 328 | 424 | 145 | 240 | 133 |
|  | 0 | 0.167 | 0.050 | 0.025 | 0.035 | 406 | 404 | 179 | 230 | 200 |
|  | -1 | -0.689 | 0.051 | 0.025 | 0.036 | 423 | 423 | 299 | 260 | 344 |
|  | -2 | -1.365 | 0.056 | 0.026 | 0.039 | 496 | 478 | 442 | 493 | 465 |
|  | -3 | -2.007 | 0.060 | 0.030 | 0.043 | 500 | 498 | 498 | 455 | 495 |
| 0.2 | 3 | 3.097 | 0.052 | 0.026 | 0.034 | 256 | 385 | 43 | 195 | 16 |
|  | 2 | 2.070 | 0.048 | 0.026 | 0.035 | 259 | 402 | 78 | 204 | 47 |
|  | 1 | 1.106 | 0.046 | 0.025 | 0.035 | 306 | 405 | 209 | 207 | 209 |
|  | 0 | 0.149 | 0.044 | 0.026 | 0.034 | 358 | 358 | 116 | 164 | 160 |
|  | -1 | -0.751 | 0.049 | 0.023 | 0.034 | 379 | 379 | 241 | 203 | 290 |
|  | -2 | -1.479 | 0.049 | 0.026 | 0.037 | 485 | 449 | 386 | 331 | 413 |
|  | -3 | -2.220 | 0.055 | 0.027 | 0.039 | 495 | 473 | 473 | 384 | 478 |

LT–*l_z_* was the number of detecting aberrant examinees by *l_z_* in 500 replications. Tables A1-A2 indicated that the |ABIAS| method outperforms the *l_z_* index, when to choose LT-68% or LT-m-68% as the judgement criterion. If we chose LT-95% or LT-m-95% as the judgement criterion, effectiveness of two methods were quite similar. If we chose LT-95% in the negative ability and LT-m-95% in the non-negative ability, the |ABIAS| method was still better than *l_z_*.

To sum up, the |ABIAS| method is better than *l_z_*, when to choose LT-68% or LT-m-68% as the judgement criterion, and it performs similarly to the *l_z_* index, when to choose LT-95% or LT-m-95% as the judgement criterion. The same results could be found in real data analysis.
